# Integrated Trauma-Focused Cognitive-Behavioural Therapy for Post-traumatic Stress and Psychotic Symptoms: A Case-Series Study Using Imaginal Reprocessing Strategies

**DOI:** 10.3389/fpsyt.2017.00092

**Published:** 2017-06-01

**Authors:** Nadine Keen, Elaine C. M. Hunter, Emmanuelle Peters

**Affiliations:** ^1^South London and Maudsley NHS Foundation Trust, Psychological Interventions Clinic for outpatients with Psychosis (PICuP), London, United Kingdom; ^2^Department of Psychology, Institute of Psychiatry, Psychology and Neuroscience, King’s College London, United Kingdom

**Keywords:** psychosis, trauma, post-traumatic stress disorder, trauma-focused cognitive-behavioural therapy for psychosis, psychological intervention, reprocessing, imaginal exposure

## Abstract

Despite high rates of trauma in individuals with psychotic symptoms, post-traumatic stress symptoms are frequently overlooked in clinical practice. There is also reluctance to treat post-traumatic symptoms in case the therapeutic procedure of reprocessing the trauma exacerbates psychotic symptoms. Recent evidence demonstrates that it is safe to use reprocessing strategies in this population. However, most published studies have been based on treating post-traumatic symptoms in isolation from psychotic symptoms. The aims of the current case series were to assess the acceptability, feasibility, and preliminary effectiveness of integrating cognitive-behavioural approaches for post-traumatic stress and psychotic symptoms into a single protocol. Nine participants reporting distressing psychotic and post-traumatic symptoms were recruited from a specialist psychological therapies service for psychosis. Clients were assessed at five time points (baseline, pre, mid, end of therapy, and at 6+ months of follow-up) by an independent assessor on measures of current symptoms of psychosis, post-traumatic stress, emotional problems, and well-being. Therapy was formulation based and individualised, depending on presenting symptoms and trauma type. It consisted of five broad, flexible phases, and included imaginal reprocessing strategies (reliving and/or rescripting). The intervention was well received, with positive post-therapy feedback and satisfaction ratings. Unusually for this population, no-one dropped out of therapy. Post therapy, all but one (88% of participants) achieved a reliable improvement compared to pre-therapy on at least one outcome measure: post-traumatic symptoms (63%), voices (25%), delusions (50%), depression (50%), anxiety (36%), and well-being (40%). Follow-up assessments were completed by 78% (*n* = 7) of whom 86% (*n* = 6) maintained at least one reliable improvement. Rates of improvements following therapy (average of 44% across measures post therapy; 32% at follow-up) were over twice those found during the waiting list period (19%). No participant indicated a reliable worsening of any symptoms during or after therapy. The study shows that an integrative therapy incorporating reprocessing strategies was an acceptable and feasible intervention for this small sample, with promising effectiveness. A randomised controlled trial is warranted to test the efficacy of the intervention for this population.

## Introduction

There are very high rates of trauma in individuals presenting with psychotic symptoms. In their review of more than 40 studies (1), Grubaugh et al. found rates of 49–100% (depending on population and methods of assessment), with 75–98% of those exposed to trauma reporting multiple traumas. Post-traumatic stress symptoms are reported in approximately 33% of people with psychosis, and 12.5% of patients with a psychotic disorder meet criteria for a diagnosis of post-traumatic stress disorder (PTSD) (2). Post-traumatic symptoms are frequently intertwined with psychotic symptoms (3, 4), with an overlap between symptoms such as hallucinations and trauma sequelae such as dissociation (5, 6). Recent theoretical models have identified underlying factors through which trauma is related to the development and maintenance of positive symptoms (7), with specific mechanisms linking different types of adversity with hallucinations and with delusions (8, 9). It is suggested that there is a direct link between the re-experiencing and arousal symptoms of PTSD, such as intrusions (“flashbacks”), and both the occurrence and content of hallucinations (9). Dissociative processes and disrupted contextual integration at the time of the trauma leave an individual vulnerable to experiencing anomalous experiences. If the trauma-memory-related intrusion is, for example, of the abuser, in combination with high levels of arousal, this may be experienced as an auditory hallucination of the voice of the abuser in individuals with an information processing style prone to weakened contextual integration (10). Further, interpersonal traumas lead to maladaptive schema and appraisals of self and others, predisposing to paranoia and delusional beliefs (11, 12). Maladaptive schema about self and others are also closely related to the concept of metacognitive capacity (13), i.e., an integrated representation of the self and others, which is suggested to be a foundation for resilience (14). Both individuals with psychosis and with PTSD show poor metacognitive mastery, which is further associated with greater distress and hyperarousal in PTSD patients (15), suggesting that a limited understanding of mental states, especially others’ emotions (16), may be another possible shared mechanism between psychotic and post-traumatic stress symptoms.

The presence of PTSD symptoms results in worse outcomes and long-term distress for many clients with trauma histories, including a poorer response to antipsychotic medication and more severe psychotic symptoms (17), higher rates of emotional disorders, substance abuse, self-harm, and suicide; and increased and longer-term use of services (1). Thus, a failure to treat trauma sequelae is costly to both service users and the National Health Service (NHS). Despite this, in clinical practice, PTSD symptoms are usually overlooked in such clients (18). Service users (19–21) have long campaigned to have professionals recognise the central role of trauma in people’s lives, and that the impact of trauma is not treated by antipsychotic medication alone. However, despite these calls, the psychological effects of trauma in those with psychotic symptoms are often not treated in mainstream services. Encouragingly, however, in the UK, the National Institute for Health and Care Excellence (NICE) (22) have, for the first time, recommended that people presenting with a first episode of psychosis in early interventions for psychosis (EIP) services should be assessed for a history of trauma and PTSD, and that NICE guidelines for PTSD (23) should be followed in those showing signs of PTSD.

Although cognitive-behavioural therapy (CBT) is recommended for both psychosis [CBT for psychosis: CBTp (22), and for PTSD (trauma-focused CBT, tf-CBT) (23)], evidence is lacking on specific, integrated tf-CBT for psychosis (tf-CBTp). CBTp does not focus directly on trauma sequelae, and therapists are often reluctant to treat PTSD symptoms due to concerns that the experiential reprocessing of trauma may exacerbate psychotic symptoms (24, 25). Consequently, this leads to people with psychotic symptoms failing to access trauma-based therapies, and/or therapists choosing modes of therapy for the trauma that exclude imaginal reprocessing techniques, which may mean that the treatment is not optimally effective. Similarly, these concerns have excluded people with psychosis (both current of a history of psychosis) from all prominent PTSD trials (26). Encouragingly however, three recent reviews (27, 28), including a Cochrane review (29), have found that there is some good quality, albeit limited, evidence to suggest that trauma-focused psychological therapies can be safe and efficacious in individuals with psychosis. Until recently, most of the studies of trauma-focused therapy in psychosis consisted of case studies (30–33), open trials (34, 35), pilot studies (36), and small-scale randomised controlled trials (RCTs) (37–39). Overall, the results are promising in terms of reduction in post-traumatic symptoms. Nevertheless, some of these studies have been in clients with “severe mental illness (SMI)” with mixed diagnosis samples, where the majority did not have psychotic disorders (39), or samples who did not necessarily present with current psychotic symptoms (38). More recently, there have been two larger-scale RCTs of trauma-focused therapy in psychosis samples. The largest was carried out in the Netherlands, and found large effect sizes for both exposure (0.78) and EMDR (0.65) on PTSD symptoms, with 57% in the exposure treatment group (*N* = 53) and 60% in the EMDR treatment group (*N* = 55) achieving a loss of PTSD diagnosis compared to 6% of the waiting list group (*N* = 47) (40). Furthermore, treatment effects were maintained 6 months later for both treatment groups. This promising outcome contrasts with the findings from a recent RCT in the UK (41), which, despite showing that tf-CBT was safe and feasible, found no differences between the therapy and treatment-as-usual (TAU) groups on PTSD outcomes. One possible explanation for these discrepancies in results is that the latter trial, unlike the Dutch study, did not include a direct memory reprocessing element in the therapy protocol but focused instead on cognitive restructuring alone.

It is, therefore, an exciting time for the development of interventions for post-traumatic stress in people presenting with psychotic symptoms. However, there is a still a lack of consensus as to the best and safest approach (e.g., cognitive restructuring versus more directive imaginal reprocessing methods such as EMDR, exposure, or re-scripting) for this vulnerable client group. In addition, there has been mixed implementation across the studies on the use of a stabilisation or preparatory phase prior to beginning the trauma-focused components of the intervention. The debate over the safety and tolerability of exposure-based PTSD therapies is not limited to those with psychotic symptoms. Therapists have long had reservations about exposure-based therapies in terms of their potential to destabilise clients (24, 25, 42), and indeed some studies have found that even during beneficial exposure-based interventions clients can experience initial worsening of PTSD symptoms, physical symptoms of anxiety, and emotional exhaustion (43–45). Thus, whilst exposure-based treatment for PTSD is effective, it can also be distressing and difficult for clients to undertake. Nevertheless, a recent review suggests that there is insufficient evidence to support a phase-based treatment approach to complex PTSD, i.e., specifically in relation to a stabilisation phase, and that its inclusion may in itself act as a delay or barrier to intervention targeting the trauma (46). Importantly, in the Dutch trial, which did not include a stabilisation phase, there were no occurrences of severe adverse events and PTSD symptom exacerbation induced by the intervention at the end of treatment or at follow-up, or of psychotic symptom exacerbation or increased suicidality (40, 47). Nevertheless, as psychotic symptom exacerbation was only measured after the first two exposure sessions (47), and current psychotic symptoms were only present in 55% of their sample, additional research is needed to build on this evidence base to gain clarity regarding the safety and efficacy of implementing exposure-based interventions within this population.

In addition, most of the studies to date have treated post-traumatic symptoms in isolation from psychotic symptoms. This may be reflective of the fact that RCTs, whilst clearly the gold standard in informing evidenced-based practice, are often required to have strict exclusion criteria, pre-defined primary outcomes, and a focus on one underlying mechanism. It is perhaps unsurprising then that despite promising outcomes in terms of PTSD symptoms, there are inconsistent results in terms of whether (and which) psychotic symptoms improve following therapy (36, 48). In clinical practice, this may pose an issue for clients whose primary goal is to address their psychotic symptoms (e.g., to reduce compliance with commanding voices) even though post-traumatic symptoms may be fuelling these, or clients who initially present and seek help for a “psychotic” explanation of their difficulties (i.e., the persecutor is following me and trying to kill me) as opposed to a “trauma” explanation (i.e., I am experiencing flashbacks of the persecutor assaulting me). This perhaps highlights the need for “practice-based evidence” integrating treatment approaches for post-traumatic stress and psychotic symptoms, and which can be tailored to the individual needs and goals of the client, in addition to the evidence base from RCTs.

The aims of the current study were to assess the acceptability, feasibility, and preliminary effectiveness of integrating current cognitive-behavioural approaches for post-traumatic stress and psychotic symptoms into a single protocol, within a routine clinical service. The therapy protocol included exposure-based reprocessing strategies since, particularly in view of recent trials (40, 41), this is likely to be a crucial mode of action in the treatment of PTSD. The protocol also includes a stabilisation phase because (1) although emotional arousal is key in exposure therapy, high levels of affect arousal in the absence of effective coping strategies may trigger information processing difficulties and psychotic symptoms (49) and (2) clinically we have found the stabilisation phase helpful in fostering an initial sense of hope and control over symptoms, and in enhancing engagement and increasing trust in the therapist. The study reports the outcomes of nine consecutive clients, obtained as part of the service’s routine assessments at baseline, pre-therapy, mid-therapy, post-therapy, and at a minimum of 6-month follow-up. The study aimed to explore changes on a range of meaningful clinical outcomes, namely measures of post-traumatic stress, psychosis symptoms, affective problems, and emotional well-being, following receipt of tf-CBTp.

## Materials and Methods

### Service Setting

These data were collected at the Psychological Interventions Clinic for outpatients with Psychosis (PICuP), based in South London and Maudsley (SLaM) NHS Foundation Trust. PICuP is a stand-alone psychological therapies clinic offering CBTp for outpatients with distressing positive symptoms of psychosis (regardless of diagnosis), or with emotional difficulties in the context of a history of psychosis. Therapists liaise closely with care coordinators in recovery multidisciplinary teams, but are not part of the team, or with general practitioners (GPs) if the person is held in primary care; they do not prescribe medication or care coordinate/case manage. Further information about the service setting, therapy and supervision structures, population characteristics, and clinical outcomes can be found in the study by Peters et al. (50).

### Ethical Approval

Ethical approval for the use of outcome data from the PICuP Clinic was obtained by the London-Dulwich Research Ethics Committee (Reference 15/LO/1831), and all participants gave written informed consent for their clinical data to be used in research studies.

### Design

A single case-series A–B design was used with five assessments (baseline, pre-intervention, mid-intervention, post-intervention, and follow-up).

### Participants

Nine participants were recruited from consecutive referrals to PICuP according to the following criteria: (1) the presence of at least one current psychotic symptom [as identified by the Psychotic Symptom Rating Scales (PSYRATS) (51) at the assessment stage]; (2) reporting distressing post-traumatic stress symptoms [scores in the moderate or above range (≥11) on the Post-traumatic Diagnostic Scale (PDS) (52)]; (3) willingness to address trauma sequelae in therapy.

The sample consisted of five (56%) men and four (44%) women, with a mean age of 37 years [standard deviation (SD) = 11.34; range 17–52]. Participants were from several ethnic backgrounds with over three-quarters (*n* = 7; 78%) from Black and Minority Ethnic (BME) groups. Seventy-eight percent (*n* = 7) were single. A substantial majority (*n* = 8; 89%) were unemployed. All participants were prescribed anti-psychotic medication. Five participants (56%) had a primary schizophrenia spectrum diagnosis (ICD-10 F20–F29), two (22%) had a primary diagnosis of PTSD (F43.1), and two (22%) had a primary diagnosis of severe depressive episode with psychotic features (F32.3). Two patients (22%) also had secondary PTSD diagnoses. The four patients with non-schizophrenia primary diagnoses did not differ from the rest of the sample in terms of presenting symptoms, and all reported persecutory delusions and hallucinations (three with command hallucinations, and three with hallucinations in multiple modalities; Table 1).

**Table 1:** **Summary of trauma type, PTSD, psychotic symptoms, target hotspot and associated memory, and key tf-CBTp interventions**.

Participant (diagnosis)	Trauma type	Psychotic symptoms	Target hotspot and associated memory	Key tf-CBTp; phase 4 intervention
**P1** *Primary*: unspecified non-organic psychosis *Secondary*: PTSD	Childhood; multiple Sought asylum in the UK	Auditory command hallucinations Visual hallucinations Paranoia	Witnessing murder of childhood friend; childhood physical abuse; held captive and tortured	Cognitive restructuring Reliving with CR Imagery rescripting (for nightmares and visions)

**P2** *Primary*: schizophrenia *Secondary*: severe depressive episode with psychotic symptoms	Adult; single event	Auditory command hallucinations Persecutory beliefs	Living in conflict zone and held at gunpoint by terrorists	Cognitive restructuring Reliving with CR

**P3** *Primary*: other non-organic psychotic disorder *Secondary*: PTSD	Childhood; multiple Sought asylum in the UK	Auditory hallucinations Visual, tactile, and olfactory hallucinations Persecutory beliefs	Living in conflict zone (civil war/genocide); parents killed. Held captive and gang raped; witnessed murder of grandfather	Cognitive restructuring Imagery rescripting

**P4** *Primary*: unspecified non-organic psychosis *Secondary*: none	Childhood; single event	Auditory command hallucinations Visual and tactile hallucinations Persecutory beliefs	Rape in childhood	Cognitive restructuring Imagery rescripting for trauma event and visual hallucinations Schema work

**P5** *Primary*: PTSD*Secondary*: emotionally unstable personality disorder	Adult; multiple	Auditory command hallucinations Visual, olfactory, and tactile hallucinations Persecutory beliefs	Several rapes and extreme physical assaults (long-term domestic violence); stillbirth of child	Cognitive restructuring Reliving with CR for trauma events Imagery rescripting for visual, olfactory tactile, and hallucinations

**P6** *Primary*: severe depressive episode with psychotic symptoms *Secondary*: none	Childhood; single event	Auditory hallucinations Persecutory beliefs	Physical and emotional abuse	Imagery rescripting Schema work

**P7** *Primary*: schizophrenia *Secondary*: severe depressive episode with psychotic symptoms	Adult; single event	Auditory hallucinations Visual, tactile, and olfactory hallucinations Persecutory beliefs	Being threatened with a knife	Cognitive restructuring reliving with CR

**P8** *Primary*: PTSD *Secondary*: severe depressive episode with psychotic symptoms	Adult; multiple Sought asylum in the UK	Commanding voices Visual and tactile hallucinations Persecutory beliefs	Long-term imprisonment with physical and sexual torture. Witnessing murder in prison	Cognitive restructuring reliving with CR Imagery rescripting

**P9** *Primary*: severe depressive episode with psychotic symptoms *Secondary*: none	Childhood; multiple	Auditory command hallucinations Visual and tactile hallucinations Persecutory beliefs	Repeated childhood physical abuse	Cognitive restructuring Imagery rescripting for trauma events, visual and tactile hallucinations, and nightmares

### Measures

The assessments consisted of a battery of measures assessing current symptoms of psychosis, post-traumatic stress, affective problems, and general well-being. The choice of routine outcome measures selected by the service is reflective of the wide range of problems held by many clients attending PICuP, and the individualized nature of therapy and people’s goals (50, 53). Pragmatic considerations typical of routine clinical services, such as financial constraints or trust-wide initiatives, led to the discontinuation of some measures [Beck Depression Inventory (BDI) and Beck Anxiety Inventory (BAI) (54, 55)], and the introduction of others (Clinical Outcomes in Routine Evaluation-10; CORE-10 (56); and Depression Anxiety Stress Scales (DASS) (57)). Hence, some participants in this study completed different measures for their affective problems and only 67% completed the CORE-10 (56).

#### The Post-traumatic Diagnostic Scale (52)

The PDS was used as a self-report screen of post-traumatic stress symptoms severity. The measure includes 17 items rated on a 4-point ordinal scale (0–3); yielding total symptom scores ranging between 0 and 51. It has been shown to have high internal consistency, test–re-test reliability, and high diagnostic validity when compared to the Structured Clinical Interview for DSM IV, and good sensitivity and specificity (52).

#### Psychotic Symptom Rating Scales (51)

This scale is a semi-structured clinician administered interview assessing the severity of 11 dimensions (frequency, duration, location, loudness, beliefs about origin, negative content, distress, disruption to life, and control) of auditory hallucinations and six dimensions (preoccupation, conviction, distress, and disruption to life) of delusions via 5-point ordinal scales (0–4). Evaluation of the PSYRATS-delusion and hallucination scales indicates good inter-rater reliability (51) and concurrent validity with the Positive and Negative Syndromes Scale (58). Total scores range between 0 and 44 for auditory hallucinations and 0 and 24 for delusions.

#### Beck Depression Inventory (55) and Beck Anxiety Inventory (54)

Twenty-one-item self-report questionnaires assessing symptoms of depression and anxiety, respectively, over the past week (BAI) or 2 weeks (BDI-II) (possible range 0–63).

#### Depression Anxiety Stress Scales-21 (57)

Twenty-one-item self-report questionnaire assessing symptoms of depression, anxiety, and stress, over the past week (range of 0–42 for each subscale). For the purpose of this study, only the depression and anxiety subscales are reported, as an alternative to the BDI-II and BAI.

#### Clinical Outcomes in Routine Evaluation-10 (56)

Ten-item-self-report questionnaire assessing emotional well-being. The CORE-10 generates a total distress score, based on each item being rated from 0 to 4, with total scores ranging from 0 (low) to 40 (severe).

#### Satisfaction with Therapy Questionnaire (STQ) (59)

An amended service-specific 22-item version of the STQ (60) was administered at the end of therapy to obtain further data regarding the acceptability of the intervention. The STQ is an adaptation of Beck et al.’s (59) Patient’s Report of Therapy Session and was first used to assess satisfaction with CBTp by Kuipers et al. (61). The measure asks about progress and satisfaction with therapy, and also includes specific items assessing clients’ beliefs in the extent to which they gained CBT skills and knowledge, perceptions of the usefulness of homework tasks set, and ratings of their therapist’s attributes. Items are scored on a scale ranging from 1 to 5, with higher scores corresponding to higher satisfaction and a score of 3 reflecting a neutral or uncertain response (e.g., unsure, no progress, and indifferent). The adapted STQ has been used with clients with psychosis in a number of other studies (61, 62).

### Procedure

Participants were assessed at five different time points on all measures as part of the routine outcome assessments for the clinic (50):

- Baseline—when first referred to the service, before going on the waiting list.
- Pre-therapy – just before starting therapy after having been on the waiting list for a median of 3 months after the baseline assessment (range 0–5 months).
- Mid-therapy—median of 5 months into therapy (range 2–7 months).
- Post-therapy—median of 22 months after starting therapy (range 8–35 months); assessments were carried out within a few days or weeks of finishing therapy (range 0–58 days; median = 7 days).
- Follow-up—median of 9 months after finishing therapy (range 5–18 months).

There were two exceptions to this: clients did not complete the second assessment (pre-therapy) if the waiting list was ≤2 weeks, and the PDS (52) was only administered at pre-, post-, and follow-up assessments; both to minimize client burden. For the purpose of this study, mid-therapy scores are not reported.

Independent assessors (assistant psychologists trained in administering all the measures) conducted the assessments [NB: at both the pre-therapy assessment and end-of-therapy assessment four participants (Ps 4, 5, 7, and 8 and Ps 1, 2, 5, and 8, respectively) declined to complete the PDS with the assistant psychologist but agreed to complete it with their therapist]. Assessments lasted between 45 and 90 min and could be conducted over more than one session if necessary. Demographic information from participants was collected at the baseline assessment.

### Therapy

All clients were offered approximately 9 months of therapy, although in practice duration of therapy was flexible, according to the clinical need (see Results). Whilst clients were in therapy with PICuP, they continued to receive routine mental health care from their recovery team (such as medication and appointments with care coordinators; *n* = 6), or their GP (*n* = 3) if they had been discharged from their team, but they did not receive other psychosocial interventions.

**Figure 1:**
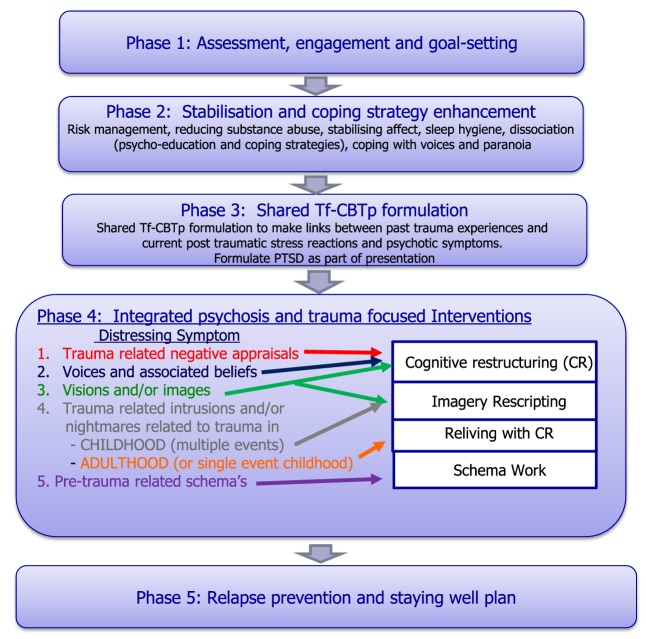
**Integrated trauma and psychosis intervention protocol**.

Therapy was usually delivered in weekly or fortnightly 60–90-min sessions. The integrated tf-CBTp therapy protocol is outlined in Figure 1, with five phases. Therapy was conducted in a flexible style with an emphasis on engagement and building a good therapeutic relationship. Although we have manualised our approach in this study, to maintain a consistent approach, in practice therapy is pragmatic in that it is adapted to the individual and their changing needs, with clinicians able to shift between stages of therapy according to clinical need. Similarly, therapy speed and progression are tailored to the individual to ensure that any changes in psychotic experiences are addressed as they arise.

Therapists consisted of two senior clinical psychologists [first (Nadine Keen) and second (Elaine C. M. Hunter) author] and one clinical psychologist (MS) supervised by Elaine C. M. Hunter. Nadine Keen and Elaine C. M. Hunter had extensive experience of delivering CBT interventions for both PTSD and for psychosis, and developed the current protocol integrating therapeutic procedures for post-traumatic and psychotic symptoms. Cases were discussed in fortnightly peer supervision groups, and MS received extra weekly individual supervision.

#### Phase 1: Assessment, Engagement, and Goal-Setting

A thorough assessment and the establishment of clients’ goals are key in determining how best to offer help. A combination of questionnaires and clinical interview is essential to maximise data gathering. Although questionnaires can feel impersonal, they are often helpful at the outset as they can feel less intrusive and normalising for clients who may be shame prone, and so clients may disclose information that otherwise they might be reluctant to say directly to their clinician. From the outset, all symptoms are framed as understandable reactions to overwhelming traumatic events and the therapist provides psychoeducation to facilitate this. To facilitate trust, engagement, and a sense of safety, a non-colluding, non-confrontational therapeutic style is required, using the client’s own terminology (63, 64). Strategies such as the symbolic panic button and having periods free from trauma/symptom discussion, to help the client work within their therapeutic “window of tolerance,” are further used as a means of sharing power and giving the client some control over the process of therapy (65). It is important to be mindful of the potential for exacerbation of affect, post-traumatic or psychotic symptoms (e.g., voices commenting, paranoia, dissociation, and flashbacks), so the pace may need to be slow with frequent checking out and reassurance. This needs to be carefully balanced with not colluding with any understandable avoidance of traumatic or affect-laden topics.

#### Phase 2: Stabilisation and Coping Strategy Enhancement

Risks of psychosis exacerbations are minimised by the inclusion of a stabilisation, coping, and affect management phase that also targets psychotic symptoms prior to trauma-focused work. Although there is debate about the inclusion of this phase (46), we find it helpful in fostering an initial sense of hope and control over symptoms, and in enhancing engagement and increasing trust in the therapist. Developing control is also helpful for the subsequent, shared formulation phase, as gaining control over some symptoms can provide evidence to support a trauma-based understanding of their experiences [e.g., “if grounding strategies stop the ‘attacks’ (i.e. somatic flashbacks) then perhaps these might be flashbacks rather than the abuser attacking me again”], and hence provides the rationale for trauma-focused interventions. Strategies employed during this phase will depend on the client’s specific presentation, needs, and goals but may include: managing and reducing risk; anxiety management (e.g., controlled breathing and special place imagery); grounding strategies; coping with voices, paranoia and other anomalous experiences; sleep hygiene and CBT strategies for depression.

#### Phase 3: Shared tf-CBTp Formulation

**Figure 2:**
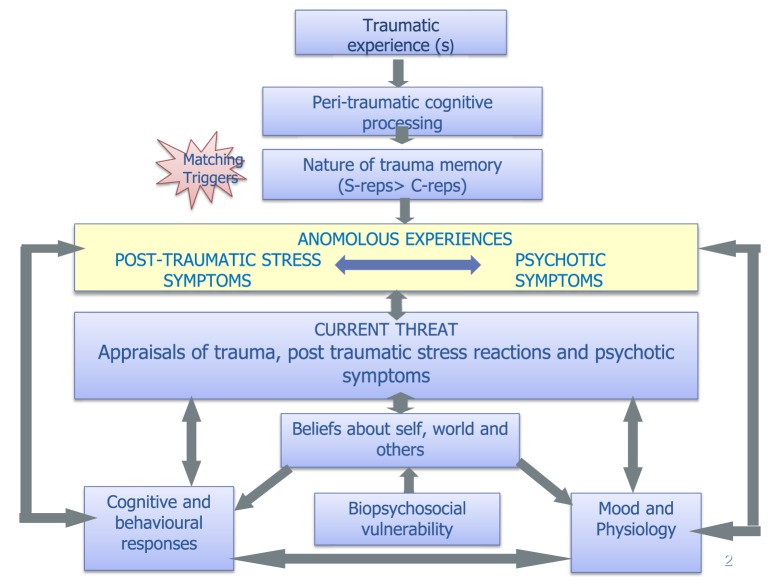
**Integrated model of trauma and psychosis [adapted from models in Ref. (3, 66, 67)]**.

By this time, the therapist may have already shared simple cognitive-behavioural maintenance vicious cycles for mood, anxiety, post-traumatic, and/or psychotic symptoms, but the aim in this study is to develop a shared formulation (template in Figure 2) helping the client to make links between their past traumatic event(s) and their current post-traumatic and psychotic symptoms. This enables possible reformulation of the client’s current difficulties as understandable responses to trauma and provides further rationale for the trauma-focused interventions. We found that changes in symptom attribution in themselves can lead to a reduction in distress. For example, P1 said he was relieved and preferred to believe the alternative explanation that his nightmares were likely caused by “unprocessed memories” of his traumatic experiences, as opposed to his original belief that they were the Devil reminding him that he was guilty of the murder of his friend. Timelines and formulation letters can also be helpful here to facilitate the contextualisation of clients’ experiences/memories and to help make links between particular events and experiences.

#### Phase 4: Integrated Psychosis and Trauma-Focused Interventions

In this phase, the psychotic and post-traumatic stress-focused interventions are listed by symptom in Figure 1 to help guide the choice of specific trauma-focused interventions. This will be guided by the formulation as well as the client’s goals and what is most distressing for them. Since trauma-related intrusions and/or nightmares are the hallmark symptoms of PTSD, we propose that the exposure element of phase 4 is an *essential* ingredient of the protocol [either imagery rescripting as described by Arntz and Weertman (68, 69) or reliving with cognitive restructuring as described by Grey et al. (70) or nightmare rescripting followed the protocol described by Sheaves et al. (71), adapted from Nappi et al. (72)]. All clients in this case series received and completed some form of exposure in their treatment (Table 1) which was preceded either by:

(1) Cognitive restructuring (*N* = 8): to reappraise negative psychosis and trauma-related appraisals including peri-traumatic hotspots (e.g., I am going to die) and/or post-traumatic appraisals (e.g., the voices tell me it was my fault, therefore it must be).
(2) Schema work (*N* = 1): when the peri-traumatic appraisal(s) (e.g., I am responsible) are rooted in congruent pre-existing schemas (which by their nature are rigid to change), it might be necessary to use more schema-focused strategies to facilitate the re-appraisal process.

#### Phase 5: Staying Well and Relapse Prevention

This phase involves the development of a relapse prevention and staying well plan, which includes a summary of key techniques and strategies learnt, as well as what to do if one faces a set-back.

### Statistical Analysis

Mean and SD are presented, and the Reliable Change Index (RCI) (73) was used as a marker of reliable change in symptoms for individual participants. Reliable change refers to the extent to which change between intervention time points falls beyond what would be expected on the basis of measurement variability. The equation uses reliability of the measure itself [using the internal consistency method for clinical populations (74), as well as a the measure of the variance of the sample (SD)]. The reliable change criterion is 1.96 times the standard error of the difference (75). If the participant falls beyond the reliable change criteria specified, it can be concluded with 95% certainty that they have shown a statistically reliable change in score, rather than that change occurring due to chance.

For the majority of measures (PDS, PSYRATS for voices and beliefs, DASS, CORE-10), the SDs and reliability scores for each measure were calculated in the software package SPSS (version 21) from baseline scores from the PICuP database (*N* = 627) of all clients seen in the service between 2003 and 2015. For the BDI and BAI, it was not possible to calculate the reliability using this method as individual item scores were not available and reliability scores were based on the data produced in a previous study (76).

Group statistics (such as paired *t*-tests) were not carried out as the sample was too small for meaningful analyses at the group level, and to minimise the number of analyses reported, bearing in mind six measures were used across three time periods [waiting list (five measures only); post-therapy; follow-up].

## Results

### Baseline Clinical Characteristics

At baseline, all participants presented with current auditory hallucinations and over half (*n* = 5; 56%) experienced command hallucinations. The majority (*n* = 7; 78%) also experienced hallucinations in other modalities (tactile, visual, somatic, or olfactory). Seventy-eight percent (*n* = 7) experienced delusions, all of which were persecutory in nature. Seventy-eight percent (*n* = 7) were in the severe or extremely severe range for depression [>28 on the BDI-II (55) or ≥28 on the DASS-21 (57)] and 89% (*n* = 8) were in the severe or extremely severe range for anxiety [>25 on the BAI (54) or ≥15 on the DASS-21 (57)]. Of the six participants who completed the CORE-10 (56), 67% (*N* = 4) fell in the moderate-to-severe or severe range for emotional well-being.

Pre therapy, 67% (*N* = 6) were in the severe range for post-traumatic stress symptoms (≥36 on the PDS (52)). Seventy-eight percent (*n* = 7) had experienced multiple traumas, 56% (*n* = 5) had experienced childhood trauma, and 33% (*n* = 3) were refugees and had sought asylum in the UK due to their traumatic experiences. Table 1 summarises the trauma type, PTSD and psychotic symptoms, target hotspot and associated memory, and key tf-CBTp interventions, for each of the participants.

### Therapy and Assessment Attrition

None of the participants dropped out of therapy. There was considerable variation across individuals in the length of therapy (median number of months = 22; range = 8–35 months) and number of sessions received (median number. of sessions = 41; range = 25–66). Towards the end of therapy, sessions were often tapered down to fortnightly, then monthly, hence prolonging therapy. Of note, one participant (P5), who received 42 sessions of the tf-CBTp protocol, also had further 82 sessions for a range of other, complex presenting difficulties, relating to secondary diagnoses of emotionally unstable personality disorder and obsessive–compulsive disorder, multiple physical health problems including frontal lobe damage following a stroke, and ongoing social needs.

**Figure 3:**
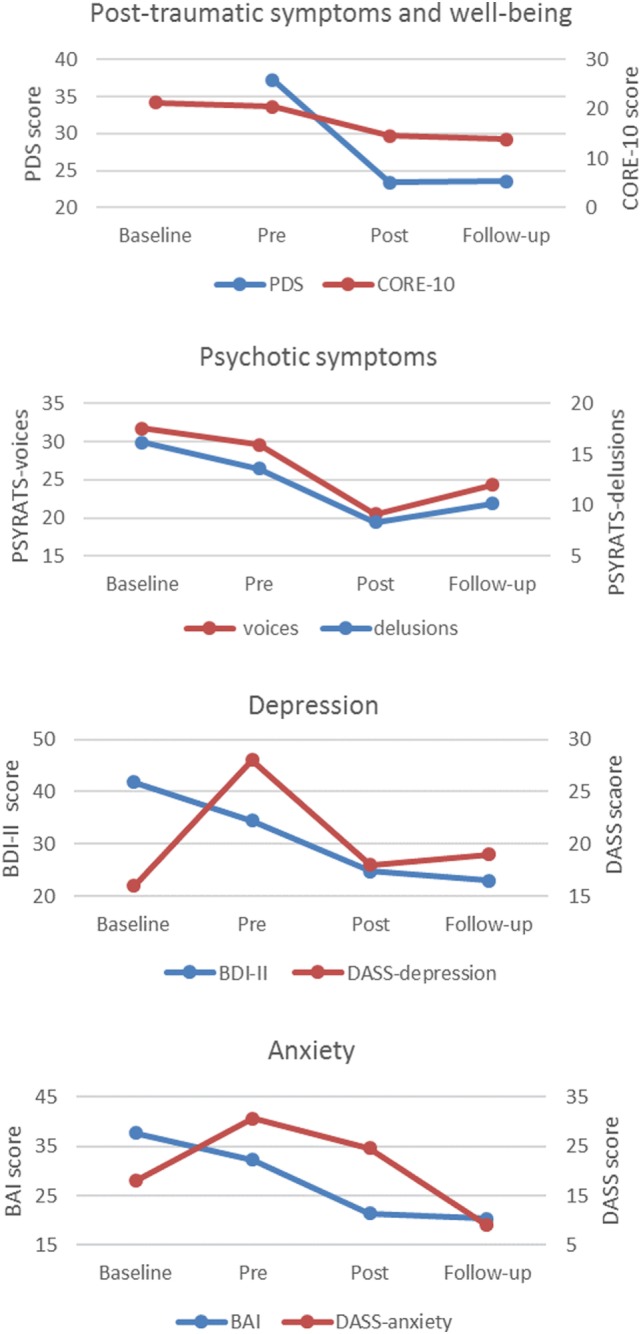
**Mean scores on outcome measures across time points**.

**Figure 4:**
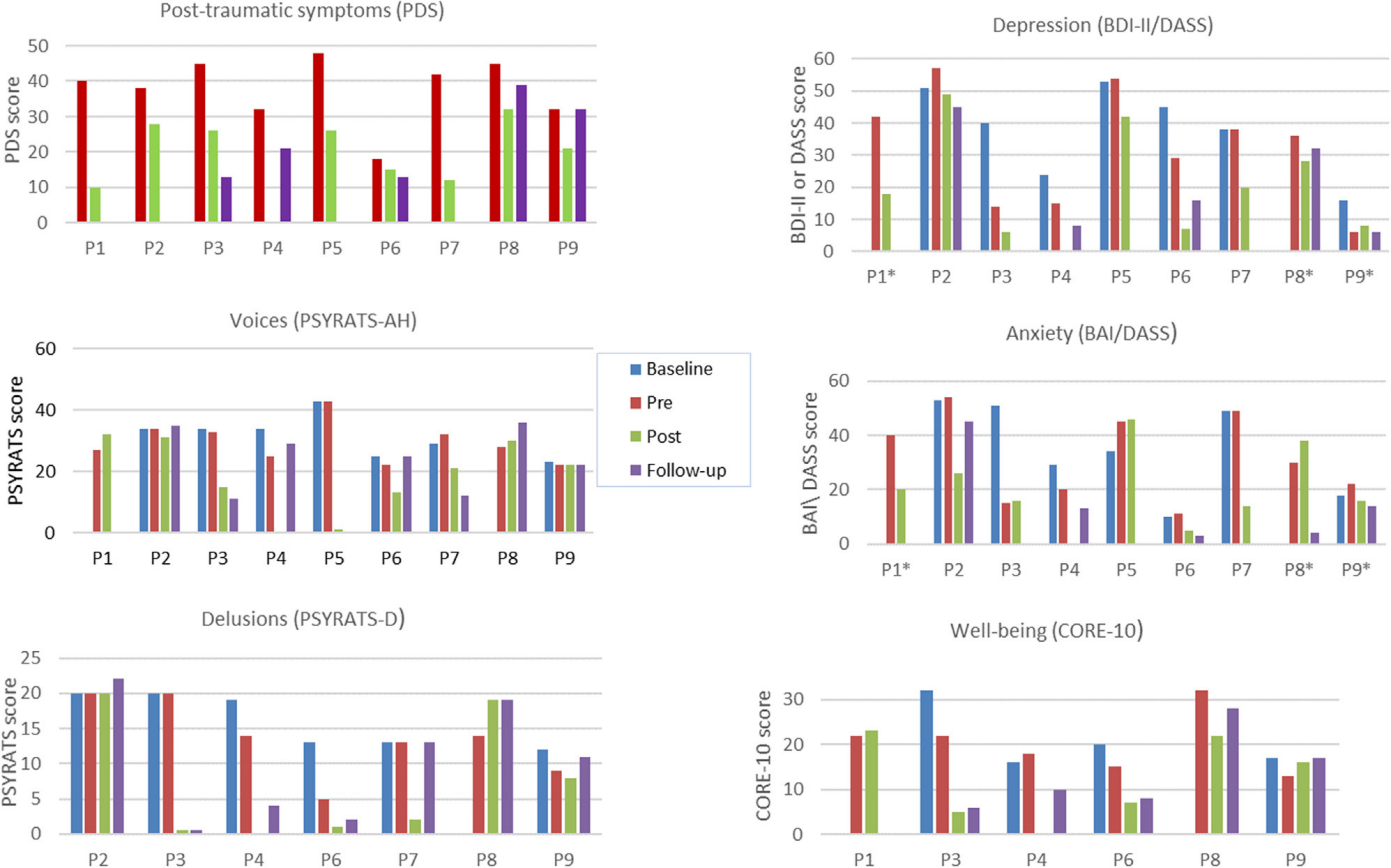
**Individual scores across time points**. Key: P, participant; * completed DASS-depression/anxiety. Missing data: Post-traumatic symptoms: baseline (all), post-therapy (P4), follow-up (P1, P2, P5, P7); Voices: baseline (P1, P8), post-therapy (P4), follow-up (P1, P5); Delusions: baseline (P8), post-therapy (P4); Depression: baseline (P1, P8), post-therapy (P4), follow-up (P1, P3, P5, P7); Anxiety: baseline (P1, P8), post-therapy (P4), follow-up (P1, P3, P5, P7); Well-being (not completed by P2, P5, and P7 at any time point): baseline (P1, P8), post-therapy (P4), follow-up (P1).

Two clients (22%; P1 and P8) did not complete the baseline assessment because the waiting list was under 2 weeks. All participants (100%) completed the pre-therapy assessment, and eight participants (89%) completed an end-of-therapy assessment. The participant (P4) who did not complete an end-of-therapy assessment completed a follow-up assessment, therefore, permitting a comparison of between pre-therapy and follow-up. Seven participants (78%) completed their follow-up assessment. One participant (P1) did not attend his follow-up appointment due to physical health issues, and one participant (P5) was not offered a follow-up because she was seen for therapy prior to this becoming a routine assessment point in the service (note that participant numbers were allocated alphabetically rather than according to referral time point). In addition, two participants did not complete measures of post-traumatic symptoms (P2 and P7), depression (P3 and P7), or anxiety (P3 and P7) at follow-up. As only seven participants experienced delusions, only 78% of the sample completed the PSYRATS-delusions (51). Only 67% (*n* = 6) completed the CORE-10 (56) as this measure was introduced in the service after three participants had already started therapy. A summary of mean data for each of the measures at each assessment point is presented in Table 2 and shown graphically in Figure 3. Individual scores on measures at each assessment point is shown in Figure 4, and a summary of changes across each phase (waiting list; pre- to post-therapy; pre-therapy to follow-up) for each measure is provided in Table 3.

**Table 2:** **Mean scores (SD; *n*) for outcomes at the four time points**.

Measure	Time point			
	Initial	Pre-therapy	Post-therapy	Follow-up
PDS	Not administered	37.22 (8.94; *n* = 9)	23.38 (12.40; *n* = 8)	23.60 (11.61; *n* = 5)
PSYRATS—delusions	16.17 (3.87; *n* = 6)	13.57 (5.44; *n* = 7)	8.33 (9.09; *n* = 6)	10.14 (8.51; *n* = 7)
PSYRATS—voices	31.71 (6.73; *n* = 7)	29.56 (6.73; *n* = 9)	20.50 (10.97; *n* = 8)	24.29 (10.06; *n* = 7)
BDI-II	41.83 (10.53; *n* = 6)	34.50 (18.60; *n* = 6)	24.80 (19.84; *n* = 5)	23.00 (19.47; *n* = 3)
BAI	37.67 (16.71; *n* = 6)	32.33 (19.05; *n* = 6)	21.40 (15.64; *n* = 5)	20.33 (21.94; *n* = 3)
DASS—depression	16 (*n* = 1)	28.00 (19.29; *n* = 3)	18.00 (10.00; *n* = 3)	19.00 (18.38; *n* = 2)
DASS—anxiety	18 (*n* = 1)	30.67 (9.02; *n* = 3)	24.67 (11.72; *n* = 3)	9.00 (7.07; *n* = 2)
CORE-10	21.25 (7.37; *n* = 4)	20.33 (6.77; *n* = 6)	14.60 (8.32; *n* = 5)	13.80 (8.96; *n* = 5)

*PDS, Post-traumatic Diagnostic Scale; PSYRATS, Psychotic Symptoms Rating Scale; BDI-II, Beck Depression Inventory-II; BAI, Beck Anxiety Inventory; DASS-21, Depression Anxiety Stress Scales-21; CORE-10, Clinical Outcomes in Routine Evaluation-10*.

**Table 3:** **Change scores for each participant’s outcome measures across phases**.

Measures (reliable change criterion for each measure)	Post-traumatic symptoms		Voice symptom levels			Delusion symptom levels			Depression			Anxiety			Emotional well-being		
	
	PDS		PSYRATS			PSYRATS			BDI			BAI; RCI = 8.83^a^			CORE		
	
	Reliable Change Index (RCI) = 12.96^a^		RCI = 9.76^a^			RCI = 7.40^a^			DASS			DASS-A*; RCI = 18.38^a^			RCI = 9.34^a^		
Time point	Pre-therapy–post- therapy	Pre-therapy–follow-up	Baseline–pre-therapy	Pre-therapy–post-therapy	Pre-therapy–follow-up	Baseline–pre-therapy	Pre-therapy–post-therapy	Pre-therapy–follow-up	Baseline–pre-therapy	Pre-therapy–post-therapy	Pre-therapy–follow-up	Baseline–pre-therapy	Pre-therapy–post-therapy	Pre-therapy–follow-up	Baseline–pre-therapy	Pre-therapy–post-therapy	Pre-therapy–follow-up

P1*	↓30	m	m	↑5	m	NA	NA	NA	m	↓24	m	m	↓20	m	m	↑1	m

P2	↓10	m	↔	↓3	↑1	↔	↔	↑2	↑6	↓8	↓12	↑1	↓18	↓9	m	m	m

P3	↓19	↓32	↓1	↓18	↓22	↔	↓20	↓20	↓26	↓8	m	↓36	↑1	m	↓10	↓17	↓16

P4	m	↓11	↓9	m	↑4	↓4	m	↓10	↓9	m	↓7	↓9	m	↓7	↑2	m	↓8

P5	↓22	m	↔	↓43	m	NA	NA	NA	↑1	↓12	m	↑11	↑1	m	m	m	m

P6	↓3	↓5	↓3	↓9	↑3	↓8	↓12	↓11	↓16	↓22	↓13	↑1	↓6	↓8	↓5	↓8	↓7

P7	↓30	m	↑3	↓9	↓20	↔	↓11	↔	↔	↓18	m	↔	↓35	m	m	m	m

P8*	↓13	↓6	m	↓2	↑8	m	↑5	↑5	m	↓8	↓4	m	↑8	↓26	m	↓10	↓4

P9*	↓11	↔	↓1	↔	↔	↑3	↓1	↑2	↓10	↑2	↔	↑4	↓6	↓6	↓4	↑3	↑4

	8 improved (5 reliably)	4 improved (1 reliably) 1 remained the same	4 improved 2 remained the same 1 worsened	5 improved (2 reliably) 1 remained the same 2 worsened	2 improved (reliably) 1 remained the same 4 worsened	3 improved (1 reliably) 3 remained the same	4 improved (3 reliably) 1 remained the same 1 worsened	3 improved (reliably) 1 remained the same; 3 worsened	4 improved (2 reliably) 1 remained the same 2 worsened	7 improved (4 reliably) 1 worsened	4 improved (2 reliably) 1 remained the same	2 improved (reliably) 1 remained the same 3 worsened	5 improved (3 reliably) 3 worsened	5 improved (2reliably)	3 improved (1 reliably) 1 worsened	3 improved (2 reliably) 2 worsened	4 improved (1 reliably) 1 worsened

*These 3 participants (P1, P8 and P9) completed the DASS-anxiety/depression as opposed to the BAI/BDI-II. *^a^RCI scores correspond to the score which had to be exceeded for a reliable change to be obtained for each particular measure*.

*Numeric figure represents change in score on each measure. Figures in red represent reliable change scores. ↓, improvement; ↔, no change; ↑, worsening; NA, non-applicable; m, missing*.

### Changes during the Waiting List Period

Data were not available for the PDS for this period. Data were also not available on other measures for the two clients (P1 and P8) who only completed a pre-therapy assessment. Means for the other seven individuals remained comparable [PSYRATS-voices; well-being (CORE-10)] or reduced marginally [PSYRATS-delusions; depression (BDI-II); anxiety (BAI)] for the majority of measures whilst participants were on the waiting list. The means remained in the severe or extremely severe ranges for depression and anxiety, and moderate to severe for emotional well-being. None of the seven participants showed a reliable improvement on PSYRATS-voices, as evidenced by the RCI. One (17%) of the six participants with delusions, and one (25%) of the four participants who completed the CORE-10, showed a reliable improvement (with 95% confidence) in delusions and well-being, respectively. Two (29%) of the seven participants showed a reliable improvement on depression and one (14%) of the seven showed a reliable improvement on anxiety (Table 3). One participant (14%) showed a reliable worsening of anxiety whilst on the waiting list. No further reliable changes were found.

### Changes during Therapy

#### Post-traumatic Symptoms

The overall mean for the group on the PDS fell from the “severe” range at pre-therapy to the “moderate-to-severe” range post therapy, which was maintained at follow-up. In terms of individual scores on the PDS, all participants completing pre- and post-assessments (*n* = 8) had lower scores post therapy, and 63% (*n* = 5) of these showed reliable improvements. No participants showed any symptom deterioration at the end of therapy compared to pre-therapy. Five (56%) participants completed the PDS at follow-up. Of these, four participants (80%) showed improvements compared to pre-therapy, one of whom (20%) showed a reliable improvement. One (20%) participant’s score on the PDS at follow-up had returned to the same level as their score pre therapy (despite showing a non-reliable improvement between pre- and post-therapy). No participants showed a worsening of post-traumatic symptoms at follow-up compared to pre-therapy.

#### Auditory Hallucinations

The overall mean for the group on the PSYRATS-voices was reduced at both post-therapy and follow-up compared to pre-therapy. Scores between pre- and post-therapy decreased in five (63%) of the eight participants who completed their post-therapy assessment, of whom two (25%) participants showed reliable improvements in their voices. Seven participants (78%) completed the PSYRATS-voices at follow-up. Of these, 29% (*n* = 2) showed reliable improvements at follow-up compared to pre-therapy. Fifty-seven percent (*n* = 4) showed a worsening in their voices, although none of these indicated reliable changes.

#### Delusions

Compared to pre-therapy, the overall mean for the group on the PSYRATS-delusions was reduced at both post-therapy and follow-up. Of the seven participants experiencing delusions, six (86%) completed the PSYRATS-delusions post therapy, and scores between pre- and post-therapy decreased in four (67%) of these, of whom three (50%) showed reliable improvements. There were no other reliable changes. All participants who experienced delusions (*n* = 7) completed this measure at follow-up. Of these, 43% (*n* = 3) showed reliable improvements in their delusion scores at follow-up compared to pre-therapy, with no other reliable changes.

#### Depression

The overall mean for the group on measures of depression fell from the severe and extremely severe ranges [on the BDI-II (*n* = 5) and DASS-depression (*n* = 3), respectively] to the moderate range, which was maintained at follow-up. Of the eight people completing their post-therapy assessment, all but one participant (*n* = 7) improved on their depression score between pre- and post-therapy, four (50%) of whom indicated reliable improvements. Five participants (56%) completed a depression measure at follow-up. Of these, 80% (*n* = 4) showed improvements in their depression scores at follow-up compared to pre-therapy, 40% (*n* = 2) of whom showed reliable improvements, with no other reliable changes.

#### Anxiety

Eight participants (89%) completed an anxiety measure at the end of therapy. The overall mean for the group on the BAI fell from the severe range pre therapy to the moderate range post therapy (*n* = 5). This was maintained at follow-up (*n* = 3). For those who completed the DASS-anxiety (*n* = 3), the overall mean scores reduced at the end of therapy compared to pre-therapy but remained in the extremely severe range. At follow-up, however, the mean for the DASS-anxiety fell to being in the mild range (*n* = 2). Sixty-three percent (*n* = 5) of participants’ anxiety improved between pre- and post-therapy, 36% (*n* = 3) of whom indicated reliable improvements. There were no other reliable changes. Five participants (56%) completed an anxiety measure at follow-up. Of these, 100% (*n* = 5) showed improvements in their anxiety at follow-up compared to pre-therapy, 40% (*n* = 2) of whom showed reliable improvements.

#### Emotional Well-being

Five participants (83%) completed the CORE-10 at the end of therapy. The overall mean for the group on the CORE-10 (*n* = 5) fell from the moderate-to-severe range at pre-therapy to the mild range at the end of therapy. This was maintained at follow-up. Four participants (80%) improved on their emotional well-being score between pre- and post-therapy, and 40% (*n* = 2) indicated reliable improvements. There were no other reliable changes. Five participants (83%) completed the CORE-10 at follow-up. Of these, 80% (*n* = 4) showed a reduction in their well-being score at follow-up compared to pre-therapy, one of whom (20%) showed a reliable improvement, with no other reliable changes.

### Summary of Change across All Symptom Measures

Visual comparison of the means between pre-therapy and the end of therapy for the whole group indicated that overall the sample showed a trend for improved scores on all symptom measures, which appeared to be maintained at 6-month follow-up (Table 3; Figure 3). At the end of therapy, all but one client (88%) achieved a reliable improvement on at least one symptom measure; 75% on at least two symptom measures; and 50% on at least three symptom measures. Of those who completed a follow-up assessment, 86% (*n* = 6) indicated at least one reliable improvement at follow-up, with 43% (*n* = 3) exhibiting a reliable change on at least two symptoms measures. No participants indicated a reliable worsening of their symptoms at either time point. One participant (14%) indicated a reliable worsening in their anxiety whilst on the waiting list. In terms of individual change scores, depending on outcome measure, there was a reduction of between 60–100% of scores from pre to post therapy, and between 29–100% of scores from pre-therapy to follow up. Reliable change was found on between 25–63% (average 44%) of scores at post therapy and between 20–43% (average 32%) of scores at follow up. This compared to a reduction in 29–75% of individual change scores in the waiting list period, with 0–29% (average 19%) of scores showing reliable improvement.

### Participant Feedback Regarding Satisfaction with Therapy

All participants (*n* = 9) completed the STQ (59) either at the end of therapy (*n* = 8) or at follow-up (P4). Overall, 78% (*n* = 7) were “very satisfied” and 22% (*n* = 2) were “satisfied” with the therapy they received. Eighty-nine percent (*n* = 8) rated that they felt they had made “a lot of progress” in therapy and one participant (11%) felt they had made “a little progress.” All participants (100%) rated that they had “a lot” of trust in their therapist; 89% felt “very understood” by their therapist; 11% (*n* = 1) felt “fairly understood” by their therapist. Eighty-nine percent (*n* = 8) endorsed that they either “strongly agreed” or “agreed” that they had achieved a better understanding of the development of their problems and 78% (*n* = 7) endorsed that they “strongly agreed” they had achieved a better understanding of their experiences. Eighty-nine percent (*n* = 8) rated that they “strongly agreed” that they had gained methods and techniques to cope with their problems. Sixty-seven percent (*n* = 6) and 33% (*n* = 3) rated that tasks between sessions were “very helpful” or “slightly helpful,” respectively. Selected verbatim comments from the nine participants regarding the intervention are presented in Table 4.

**Table 4:** **Examples of participant comments on their response to the tf-CBTp intervention**.

Selected comments from participants
“It was like being woken from a lifelong coma—I can actually start to live again.”
“I feel more in control. I still hear the voices but I don’t have to do what they say. I don’t feel I’m back there again. They are just memories from the past.”
“I never thought I would be able to say the words ‘It’s not my fault’ but I have learnt to, and I believe it. I can now move forward.”
“If you don’t talk about it, the root of the trauma is still there, it just keeps coming back and you end up in repeated vicious cycles. I wouldn’t be here today if I wasn’t referred…not only did it help me recover but it was educational and empowering.”
“I think if more people were offered trauma focused therapy there would be less mad people, or at least, less people thinking that they are mad.”
“I could write a book about my experience of therapy and I would definitely refer a friend.”
“Things I found helpful included: imagery rescripting, cross examination of evidence and alternative explanation of beliefs.”

## Discussion

This case-series study demonstrated that it is safe, feasible, and acceptable to integrate current cognitive-behavioural approaches for post-traumatic stress and psychotic symptoms into a single protocol, in individuals with current distressing symptoms of psychosis and post-traumatic stress presenting to a psychological therapies service. All participants completed therapy, and no adverse outcomes in terms of reliable worsening of symptoms, relapse, or hospital admissions occurred as a result of the intervention. Satisfaction ratings were very high, and all but one client (88%) achieved a reliable improvement on at least one symptom measure post therapy.

Visual examination of the group means showed a trend for improved scores across all symptoms, both at the end of therapy and at the 6+ months of follow-up. In terms of individual change scores, it is very encouraging that a substantial majority showed improvements on each of the symptom measures, with approximately one-third of the participants indicating reliable changes on each of the measures at each time point. Rates of improvements following therapy were twice those found during the waiting list period, for those outcomes where waiting list data were available (i.e., all measures except PDS (52)). The greatest improvement noted was on post-traumatic symptoms, in which all clients showed improved scores, with 63% indicating a reliable change. These promising findings provide some support for the view that the inclusion of direct memory reprocessing strategies is necessary to address post-traumatic symptoms.

Whilst these results are encouraging, it is important to note that the mean PDS score was still in the moderate-to-severe range post therapy and at follow-up (as compared to being in the severe range pre therapy). Nevertheless, it is worth bearing in mind that this was a very complex sample with the majority (78%) presenting with multiple complex traumas (e.g., participant 2 experienced long-term imprisonment with physical and sexual torture, and participant 3 was living in conflict zone (civil war and genocide) during which her parents were killed. She was then held captive, gang raped, and witnessed the murder of grandfather). Over half (56%) had experienced childhood trauma, and a third (33%) were refugees (who in addition to their index trauma had additionally experienced the trauma of living in conflict zones and having to relocate to the UK). The majority of the clients had ongoing social needs, multiple diagnoses, and high levels of baseline depression and anxiety (78 and 89% scoring in the severe or extremely severe range for depression and anxiety, respectively). Given these profiles, it is perhaps understandable that many clients had some residual post-traumatic symptoms post therapy (albeit much reduced).

In terms of impact on voices, the majority of participants indicated improved scores but only a minority improved reliably post therapy (25%) or at follow-up (29%). It is worth noting that in this sample, individuals’ voices were very severe, with over half (56%) experiencing distressing command hallucinations associated with risky compliance behaviours. Greater improvements were seen for delusions, with approximately half indicating reliable improvement post therapy (50%) or at follow-up (43%). This change may be explained by the fact that the therapy protocol included re-formulating distressing delusional beliefs (e.g., the Devil is suffocating me at night) as understandable reactions to traumatic experiences (e.g., feeling suffocated is a flashback to my abuser who reminds me of the Devil), hence potentially providing alternative, less distressing explanations for clients’ delusional beliefs (77). These results are consistent with the findings from the Dutch trial, where changes were found for paranoid thoughts but not hallucinations (48), although fewer of their patients presented with voices than paranoia at baseline, which may have skewed their results. Impact on affect and emotional well-being was also encouraging, and most people indicated improved scores on measures of depression, anxiety, and well-being with 50, 36, and 40% indicating reliable changes on these, respectively, many of whom maintained their scores, or at least showed some improvement at follow-up (40, 40, and 20%, respectively).

It should be noted that the intervention was long (median 41 sessions) and included a stabilisation phase, unlike in the Dutch trial (40). Clinically, it was felt that the stabilisation phase was necessary to build coping strategies, foster a sense of control, and trust in the therapist, and to provide a rationale for the trauma-focused phase of the therapy. Indeed, client feedback was positive with regard to this (e.g., “I understood why it kept coming back and by learning to control the visions I felt more empowered to tackle it head on” [P7]). This supports previous findings within the PTSD literature that therapeutic alliance during the first phase of treatment predicts successful reduction of PTSD symptoms during exposure therapy in the second phase (78). Furthermore, for the majority of the clients, there were ongoing social issues (e.g., immigration, housing, and ongoing domestic violence) that needed to be addressed prior to some clients feeling able to commence the trauma-focused phase of the treatment. As therapists were often the only mental health professionals involved in the client’s care, it frequently became therapists’ responsibility to address these issues, and, therefore, the length of therapy should be interpreted in this context. Further, given that the NICE guidelines recommend (even when treated in isolation) at least 12 sessions for PTSD, with more for complex PTSD or multiple traumas (23), and at least 16 for psychosis (22) and for severe depression (79), in addition to the recent finding that 25 sessions is the optimal dose for CBT for psychosis (80), 41 sessions is perhaps understandable for this very complex client group. However, whether the stabilisation phase is necessary remains an empirical question that needs to be tested. Consideration will need to be taken with regard to different service contexts, and how some may be better equipped than others to provide holistic care (i.e., in some community teams, e.g., care coordinators may be able to provide stabilisation strategies alongside a therapist doing the trauma-focused work).

Overall, these findings support the emerging evidence base in this area (27–29) and suggest that an integrated approach, targeting both psychotic and post-traumatic symptoms, can have a positive impact not only on positive symptoms of psychosis and post-traumatic symptoms but also on depression, anxiety, and emotional well-being for some clients. The results should be interpreted within the context of a number of strengths and limitations.

### Strengths

The study has good ecological validity in that the sample was representative of the heterogeneity and complexity of individuals presenting with distressing post-traumatic stress and psychotic symptoms in the clinic, unlike RCTs that have been criticized on the basis of strict inclusion criteria, and which include individuals without current psychotic symptoms.

The intervention was well received by participants. A number of key factors were likely involved in keeping people engaged in therapy. First, we recruited only individuals who expressed willingness in addressing their trauma sequelae. Many individuals are highly avoidant of discussing their trauma, and ascertaining readiness for therapy is crucial. Second, the therapy adhered to the central tenets and values of CBTp (64, 81): much emphasis was given to engagement and a good therapeutic relationship; therapy was collaborative, with active participation from the client who is seen as an expert in his or her experiences, and had a normalizing philosophy; it was geared towards achieving the person’s personal valued goal(s) and based on an individualized, shared formulation, which provided a rationale for trauma-focused interventions; emotional processes were fully integrated within the formulation of psychotic and post-traumatic stress symptoms, and addressed in therapy, not just as the sequelae, but in terms of their role in the formation, trigger, and maintenance of symptoms; paramount importance was given to maintaining the person’s self-esteem, empowering and providing hope, and promoting recovery-oriented values (82). Third, we felt the stabilisation stage was helpful in preparing the client for the exposure work ahead, providing hope, trust and optimism, and minimising the risk of exacerbations of psychotic symptoms. Fourth, trauma exposure was conducted within the client’s therapeutic “window of tolerance,” in that the client was in control of the amount and duration of direct PTSD exposure they could manage within the therapy sessions. This is likely to have been a key factor in minimising dropouts in the therapy process. Finally, the flexible and pragmatic nature of the protocol meant that the intervention was tailored to the needs and goals of the individual, and targeted psychotic and post-traumatic symptoms in an integrated, rather than isolated, fashion. Other groups, such as Lysaker and Dimaggio, have also recommended the use of integrated approaches for people with psychosis, rather than developing new schools of treatment, which promote guided discovery to foster an increased awareness of mental phenomena and making sense of what is happening in one’s own mind and the minds of others (i.e., targeting metacognitive capacity, a potential common mechanism in post-traumatic and psychotic symptoms), to move meaningfully towards recovery (83).

The use of independent assessors, rather than outcomes being elicited by the therapists themselves, is a further strength, although a number of participants only agreed to complete the PDS with their therapists. The availability of data from five assessment points, including during a waiting list period, meant that this could serve as a control for each participant, and allowed 6+ months of follow-up for 78% of participants.

### Limitations

The study design was limited by the small number of participants, although a sample size of nine is acceptable for a case series. Nevertheless, we were unable to report group statistics, or to investigate potential differences between those who showed improvements on specific outcomes and those who did not. Unfortunately, there were no data available for the PDS during the waiting list period. This means that it is not possible to draw firm conclusions regarding the impact of the intervention on post-traumatic symptoms. There were also missing data for some clients on certain measures or at particular time points. Due to service changes, two different measures were used for different clients to assess depression (BDI-II and DASS) and anxiety (BDI and DASS). The PDS has been shown to have a tendency to over-diagnose PTSD (84). The Clinician-Administered PTSD Scale for Schizophrenia (CAPS-S) (85) would have been a more robust, albeit more time-consuming measure. Moreover, as this is a new therapy protocol, which was being developed and elaborated over time, there were no adherence or competence ratings obtained for the therapy delivered. Also, given this was a pragmatic study in which the protocol employed a range of techniques designed to be tailored to the clients’ needs and goals, the key mechanism of change remains unclear. A further limitation was that all therapists were experienced in the delivery of this work and had assured protected time for the delivery of the therapy and attendance at supervision, free from competing demands of multidisciplinary team work. It would be important to ascertain whether it would be feasible to implement this in an NHS setting with therapists with a range of experiences, and in a range of settings.

## Conclusion

This study has important implications for the psychological treatment of post-traumatic stress symptoms in people presenting with psychotic symptoms. First, it builds on emerging evidence that treating post-traumatic symptoms through the use of imaginal reprocessing strategies is safe, acceptable, and feasible in this population. It also supports van den Berg et al.’s (40) assertion that there does not appear to be any justification for excluding people with current psychotic symptoms from trauma-focused interventions. Furthermore, it suggests that an integrated approach, targeting both psychotic and post-traumatic symptoms, can have a positive impact not only on positive symptoms of psychosis and post-traumatic symptoms but also on depression, anxiety, and well-being. An RCT is now warranted to test the efficacy of the intervention for this population. It will be important for future studies to also report on functional gains such as change in employment status or number of health care visits, as well as outcomes such as quality of life and social functioning.

## Ethics Statement

Ethical approval for the use of outcome data from the PICuP Clinic was obtained by the London-Dulwich Research Ethics Committee (Reference 15/LO/1831), and all participants gave written informed consent for their clinical data to be used in research studies.

## Author Contributions

NK: made substantial contributions to the design of the work; participated in the acquisition, analysis, and interpretation of the data; delivered the therapy for several participants (*n* = 6) in the study; took the lead for drafting and revising the manuscript; gave final approval of the version to be published; agreed to be accountable for all aspects of the work in ensuring the questions related to the accuracy or integrity of any part of the work. EH: made substantial contributions to the design of the work and the acquisition of data; delivered the therapy for participants (*n* = 2) in the study; supervised the delivery of therapy for one other case; made substantial contributions towards drafting and revising the manuscript; gave final approval of the version to be published; agreed to be accountable for all aspects of the work. EP: made substantial contributions to the conception or design of the work; participated in the analysis and interpretation of the data for the work; made substantial contributions to drafting and revising the manuscript; gave final approval of the version to be published; agreed to be accountable for all aspects of the work.

## Conflict of Interest Statement

The authors declare that the research was conducted in the absence of any commercial or financial relationships that could be construed as a potential conflict of interest.
